# Butein Interacts at the Minor Groove of DNA: Multi-Spectroscopic, Thermodynamic, and Bioinformatic Analyses

**DOI:** 10.3390/molecules31203750

**Published:** 2026-10-19

**Authors:** Jihad Alrehaili, Razique Anwer

**Affiliations:** Department of Pathology, College of Medicine, Imam Mohammad ibn Saud Islamic University (IMSIU), Riyadh 4233-13317, Saudi Arabia; jaalrehaili@imamu.edu.sa

**Keywords:** DNA, butein, drug–DNA binding, spectroscopy, molecular simulation

## Abstract

DNA is an important therapeutic target, and characterizing its interactions with small molecules can inform drug development. Butein is a chalcone with reported antioxidant, anti-inflammatory, and anticancer activities. This study investigated the interaction between butein and calf thymus DNA using spectroscopic and computational methods. UV–visible absorption and fluorescence quenching measurements supported complex formation. Fluorescence-derived binding constants ranged from 4.04 × 10^3^ to 5.62 × 10^3^ M^−1^, indicating weak to moderate binding affinity. Thermodynamic analysis indicated spontaneous binding that was both enthalpically and entropically favourable. DNA melting, urea disruption, viscosity, and competitive displacement assays supported a predominantly minor-groove binding mode. Molecular docking identified hydrogen bonds and van der Waals contacts between butein and DNA. Molecular dynamics simulations suggested that the complex remained stable over 250 ns at 310 K, with limited changes in DNA structure and dynamic hydrogen bonding. These findings characterize the interaction between butein and DNA and provide a basis for further investigation of its biological significance.

## 1. Introduction

Deoxyribonucleic acid (DNA) is the genetic material of cellular organisms and an important target for therapeutic drugs. Small molecules can interact with DNA and influence replication, transcription, and genome stability. The major and minor grooves, together with the sugar–phosphate backbone, provide several sites for non-covalent ligand binding [1]. Interactions between drugs and DNA can influence transcription and replication processes, potentially impacting various human diseases [2]. Groove binding often occurs without substantial changes in DNA structure [3]. The formation of DNA–drug complexes can induce subtle changes in both DNA and drug molecule itself. As drug–DNA interactions affect many physiological responses, detailed biophysical characterization is essential to understand the biological activity. The measurements of binding mode, affinity and thermodynamics provide mechanistic details about the role of various drugs. Hence, detailed studies on binding mode and energetics are important in drug–DNA research [4]. Calf thymus DNA was selected as a model because of its widespread use and well-documented physicochemical properties, allowing comparison with published drug–DNA binding studies.

Butein, a chalcone flavonoid, is isolated from medicinal plants such as *S. anacardium*, *T. vernicifluum*, *B. monosperma* and *D. odorifera* [5]. It exhibits diverse bioactivities including anticancer, antioxidant, antidiabetic, anti-inflammatory, antibacterial and antinephritic effects [5,6]. Butein has shown cytotoxicity against osteosarcoma, hepatocarcinoma, lymphoma and breast cancer cell lines [7,8]. Mechanistically, it can downregulate STAT3-dependent transcription and activate mitochondrial caspase-3, ultimately reducing cell proliferation [9]. Butein has also been reported to inhibit HIV-1 protease, when combined with orobol [10,11]. Evidence also indicates butein reduces advanced glycation end products and aldose reductase activity and lowers sorbitol accumulation in diabetic rat models [12,13].

Butein has been reported to induce DNA damage and fragmentation in cancer cells [14]. However, the nature of its direct interaction with DNA remains unclear. In this study, we combined in vitro experiments with computational analyses to investigate the binding mode, affinity, thermodynamics, and stability of the butein–DNA complex.

## 2. Results and Discussion

### 2.1. UV-Vis Absorption Analysis

**Figure 1:**
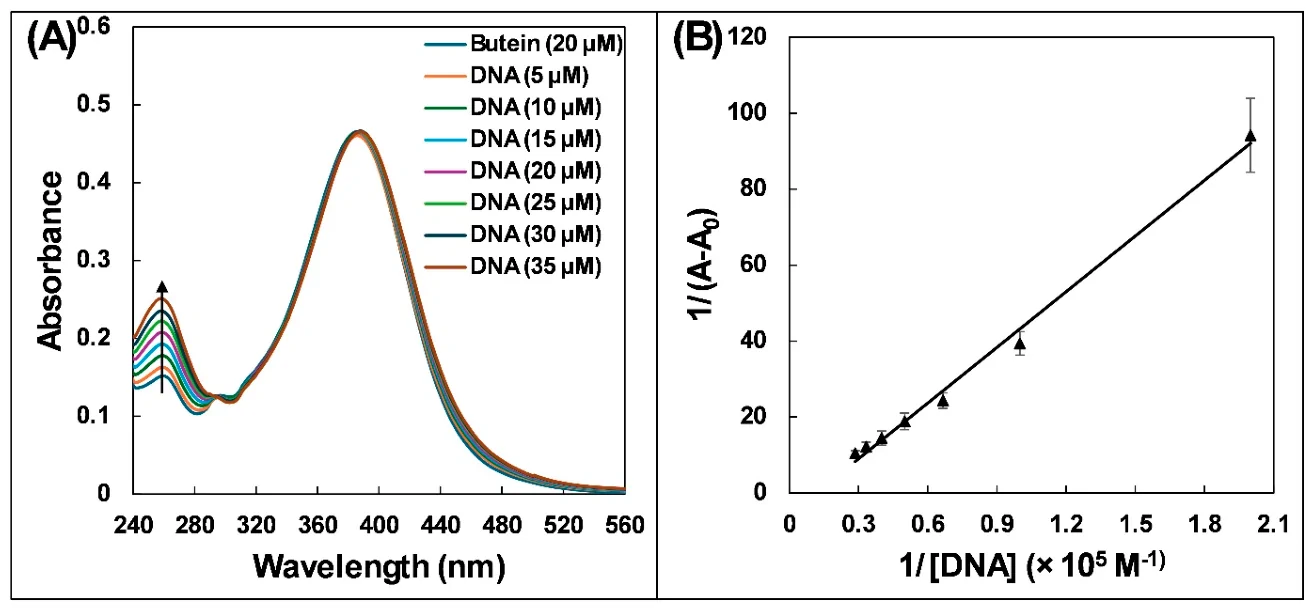
(**A**) UV-vis absorption spectra of butein (20 µM) in absence and presence of DNA (5–35 µM). (**B**) Plots of 1/(A − A_0_) versus 1/[DNA] for DNA and butein interaction. Butein = 20 µM; DNA = 5, 10, 15, 20, 25, 30, and 35 µM.

UV–visible spectroscopy was used to investigate complex formation between butein and DNA. Butein exhibited absorption peaks at 260 and 385 nm (Figure 1A), consistent with previous reports [15]. Spectra were recorded at DNA concentrations ranging from 5 to 35 μM. Because DNA also absorbs at 260 nm, the spectrum of DNA alone at each concentration was subtracted from the corresponding spectrum of the butein–DNA mixture. There were negligible changes in the absorption of butein at 385 nm. However, there was a progressive increase at the 260 nm absorption peak after DNA binding. Therefore, absorbance at 260 nm was used for calculations. The observed hyperchromicity indicates the formation of a complex between DNA and butein. For the quantitative assessment of this interaction, Equation (1) was employed [16]:

(1) $$\frac{1}{A-A_{0}}=\frac{1}{A}+\frac{1}{K_{a}\times A\times [\mathrm{DNA}]}$$

where *A*_0_ is butein’s absorbance; *A* is butein’s absorbance with DNA; *K_a_* is binding constant; [DNA] is DNA concentration. Figure 1B depicts the plot of 1/(*A* − *A*_0_) versus 1/[DNA], which shows a linear relationship. The binding constant, calculated from the intercept-to-slope ratio, was (1.16 ± 0.07) × 10^4^ M^−1^, indicating weak to moderate binding affinity between butein and DNA. The data align with a previous report in which the binding constant for the complexation of DNA with vanillin was 6.91 × 10^3^ M^−1^ at 310 K, as determined by UV-vis spectroscopy [16]. Although the values differ numerically, both of these values fall within the 10^3^–10^4^ M^−1^ range, which is typical for weak to moderate groove binders, and such variation is expected due to differences in ligand structure and assay conditions.

### 2.2. Fluorescence Quenching Analysis

**Figure 2:**
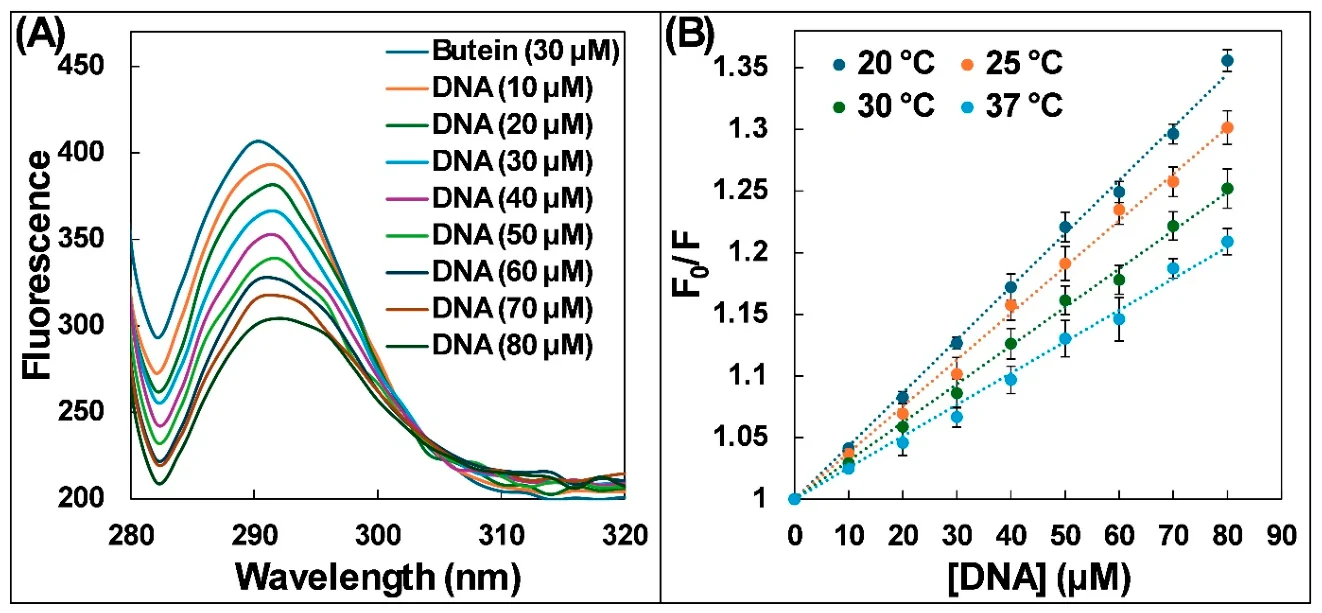
(**A**) Fluorescence emission spectra of butein (30 µM) in absence and presence of DNA (10–80 µM). (**B**) Stern–Volmer plot of F_0_/F versus [DNA] for DNA and butein interaction. Butein = 30 µM; DNA = 10, 20, 30, 40, 50, 60, 70, and 80 µM.

To further examine the interaction, we employed steady-state fluorescence spectroscopy. A fixed butein concentration (30 μM) was excited at 260 nm, and fluorescence signal was recorded between 280 and 350 nm. UV–visible spectra recorded before and after irradiation at 260 nm showed no appreciable changes. This observation supports the photostability of butein under the fluorescence measurement conditions. Since DNA also absorbs at 260 nm, the observed fluorescence (*F_obs_*) was corrected for inner-filter effects using the equation *F_corr_* = *F_obs_* × 10^(*Aex* + *Aem*)/2^. Butein exhibited an emission maximum at 290 nm. Increasing DNA concentration progressively decreased the fluorescence intensity and produced a slight red shift in the emission maximum (Figure 2A). Measurements were performed at 293, 298, 303, and 310 K to assess temperature-dependent quenching and determine the binding thermodynamics. This temperature range was selected because it spans from near-ambient laboratory conditions (293 K) to physiological temperature (310 K), thereby allowing assessment of the stability and thermodynamics of the DNA–butein complex under biologically relevant conditions. This quenching of fluorescence intensity suggests the interaction between butein and DNA. For quantitative analysis, we utilized the Stern–Volmer Equation (2). The ratio of butein’s fluorescence without and with DNA (F_0_/F) was plotted against the DNA concentration ([DNA]).

(2) $$\frac{F_{0}}{F}=1+K_{\mathrm{sv}}\left[\mathrm{DNA}\right]$$

where F_0_ is butein’s fluorescence; F is fluorescence of the DNA–butein complex; and K_sv_ is the Stern–Volmer constant. Stern–Volmer plots at the four temperatures are presented in Figure 2B. The slope of this linear plot at fixed intercept (*y*-axis intercept = 1) provides K_sv_. The K_sv_ values obtained were in range of 10^3^ M^−1^, as shown in Table 1. The Stern–Volmer plots demonstrate fluorescence quenching but do not, by themselves, distinguish between static and dynamic mechanisms [17].

To obtain further insights into the specific type of quenching (static or dynamic), we calculated the quenching constant (K_q_) using Equation (3):

(3) $$K_{q}=\frac{K_{\mathrm{sv}}}{\tau_{0}}$$

where τ_0_ is the assumed fluorescence lifetime of unquenched butein (~10^−8^ s) [18]. The analysis revealed that K_q_ values, listed in Table 1, fall in the range of 10^11^ M^−1^ s^−1^. This value is significantly higher than the limiting diffusion rate constant [19]. The estimated K_q_ values were consistent with a static quenching mechanism. Another approach to distinguish between static and dynamic quenching is to analyse K_sv_ at different temperatures (Table 1). K_sv_ values decreased as the temperature increased. This observation aligns with the characteristics of static quenching [20]. For static quenching, where a complex forms, higher temperatures generally destabilize the complex. The destabilization leads to lower quenching efficiency, resulting in a decrease in K_sv_ with the increase in temperature. In dynamic quenching, an increasing K_sv_ value is found with rising temperature due to more frequent collisions at higher temperatures [20].

### 2.3. Determination of Binding Constants and Thermodynamic Parameters

**Figure 3:**
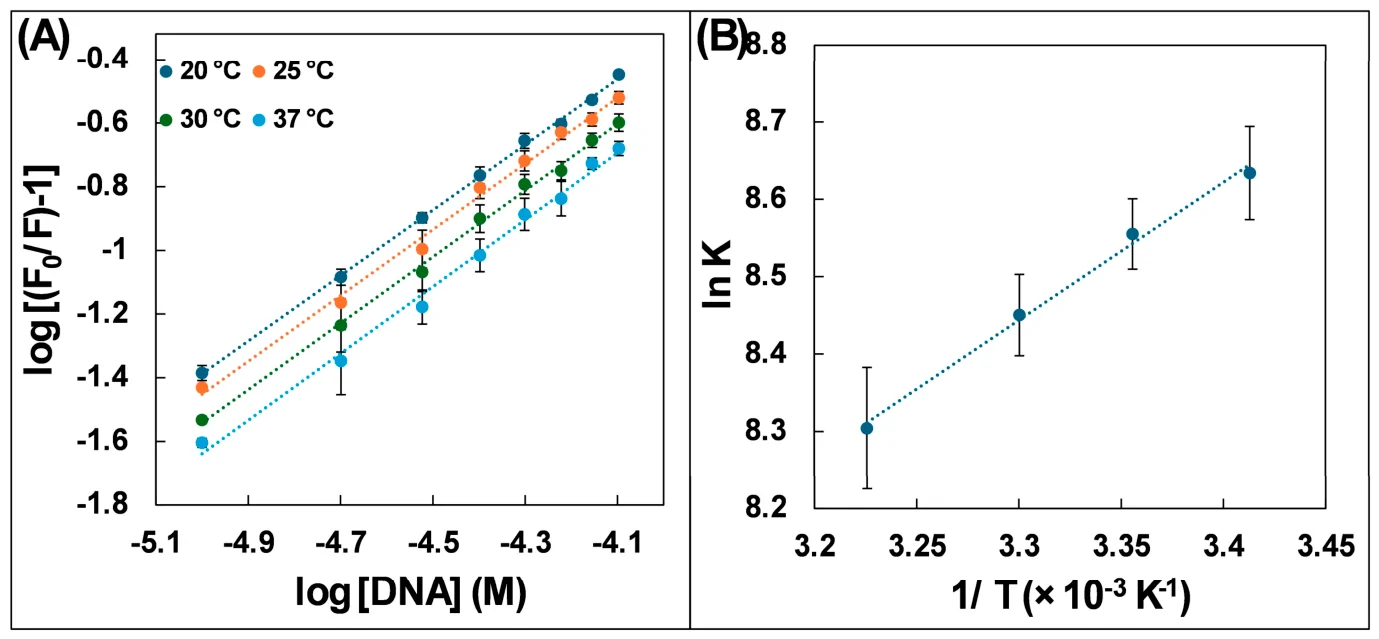
(**A**) Modified Stern–Volmer plot of log [(F_0_/F) − 1] versus log [DNA] for DNA and butein interaction. Butein = 30 µM; DNA = 10, 20, 30, 40, 50, 60, 70, and 80 µM. (**B**) van’t Hoff plot of ln K versus 1/T for DNA–butein interaction.

The data obtained from fluorescence quenching assays were further used to calculate thermodynamic parameters and binding constants. Equation (4) was used for the calculation of binding constants (K).

(4) $$\log[\frac{F_{0}}{F}-1]=\mathrm{log K}+\mathrm{n log}\left[\mathrm{DNA}\right]$$

where n represents the number of binding sites, and K is the binding constant. Using log plot of log [(F_0_/F) − 1] vs. log [DNA], we can determine the values of n and K. As illustrated in Figure 3A, the intercept of log plot yields log K, while slope yields n. The binding constants at all four temperatures were in the order of 10^3^ M^−1^ (Table 2). The values are close to the binding constant obtained using UV–visible spectroscopy. Although the directly measured binding constants are lower than those of common groove binders, such affinities can still be biologically relevant because cellular effects depend on local concentration and cooperative interactions.

Thermodynamic parameters that influence the nature of intermolecular forces in DNA complex formation are entropy change (ΔS), free energy change (ΔG), and enthalpy change (ΔH) [21]. The van’t Hoff Equation (5) was used to calculate ΔH and ΔS using fluorescence quenching data [21].

(5) $$\ln K=-\frac{\Delta H}{\mathrm{RT}}+\frac{\Delta S}{R}$$

In above equation, R is universal gas constant (1.987 cal K^−1^ mol^-l^), and T is the temperature. The van’t Hoff plot of ln K vs. 1/T was used to calculate ΔS and ΔH values, and the plot is presented in Figure 3B.

The ΔG was calculated using Equation (6):

ΔG=ΔH − TΔS(6)

Table 3 summarizes thermodynamic parameters for DNA and butein interaction. Analysis of these values, particularly their signs and magnitudes, offers insights into the nature of intermolecular forces [22]. Generally, various non-covalent interactions like hydrophobic forces, hydrogen bonds, electrostatic interactions, and van der Waals forces are involved in such biomolecular interactions [22]. The negative ΔG values indicated spontaneous binding under the experimental conditions, while the negative ΔH values indicated an exothermic interaction [16].

### 2.4. Assessment of DNA Melting Studies

**Figure 4:**
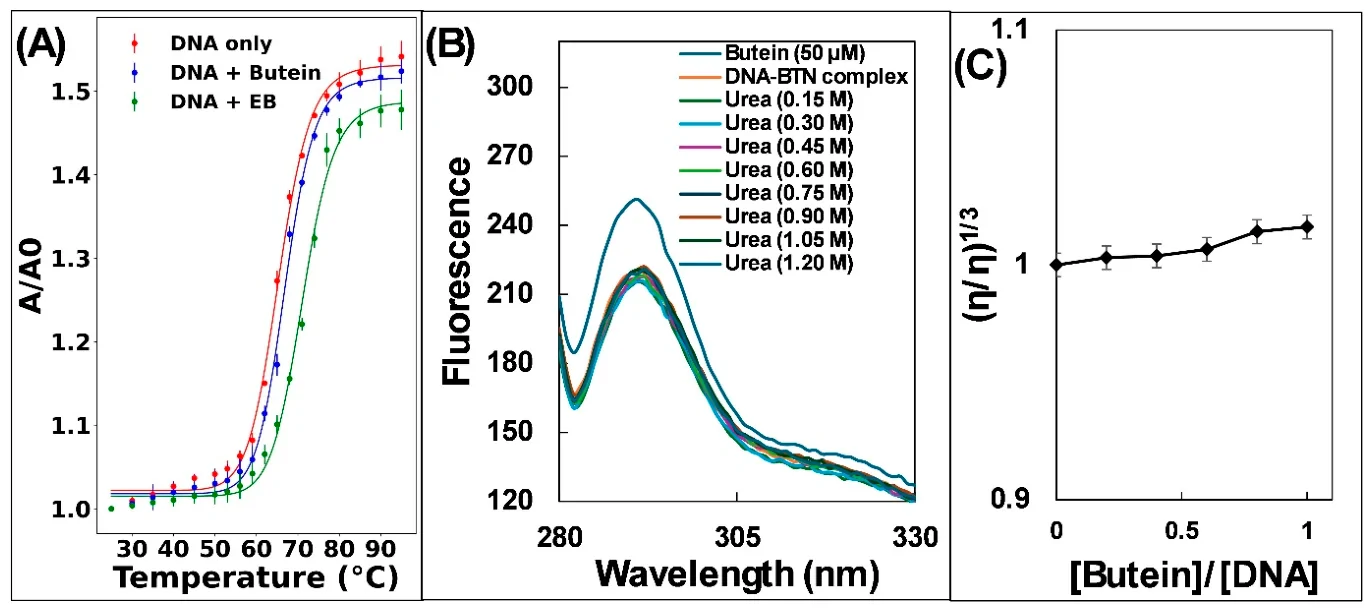
(**A**) Thermal denaturation curve of DNA in absence and presence of butein or ethidium bromide. (**B**) Effect of urea on fluorescence emission spectrum of DNA–butein complex. Butein = 50 µM; DNA–butein complex = 50 µM each; urea = 0.15, 0.30, 0.45, 0.60, 0.75, 0.90, 1.05, and 1.20 M. (**C**) Effect of butein interaction on the viscosity of DNA. DNA 100 µM; butein = 20, 40, 60, 80, and 100 µM.

The DNA double helix is stabilized by hydrogen bonds between complementary bases and stacking interactions between adjacent bases. Heating disrupts these interactions and leads to strand separation. The melting temperature (Tm) is the temperature at which half of the DNA is denatured [23]. Interestingly, small molecules like drugs can influence DNA’s T_m_. Molecules that intercalate between base pairs, such as EB, enhance DNA stability and typically raise T_m_ by 5–8 °C [24]. In contrast, groove binding or electrostatic interactions of drugs with DNA often have minimal impact on T_m_. The T_m_ of free DNA was 65.45 ± 0.21 °C, while the DNA–butein complex showed T_m_ of 66.99 ± 0.10 °C (Figure 4A). However, T_m_ of the DNA-EB complex was 71.16 ± 0.17 °C. The minimal T_m_ change observed between free DNA and the DNA–butein complex (1.53 °C) suggests that butein may be a DNA groove binder.

The denatured fraction (α) was calculated from the absorbance data, and the apparent equilibrium constant was calculated as K = α/(1 − α). The slope and intercept of the plot of ln K against 1/T were used to estimate ΔH and ΔS, respectively. ΔG was subsequently calculated at 298 K. The results show ΔH = 32.21 kcal/mol, ΔS = 96.01 cal mol^−1^ K^−1^, and ΔG = 3.59 kcal/mol at 298 K for DNA alone. In the presence of butein, ΔH = 32.81 kcal/mol, ΔS = 97.26 cal mol^−1^ K^−1^, and ΔG = 3.81 kcal/mol at 298 K, while EB binding produced ΔH = 35.56 kcal/mol, ΔS = 104.69 cal mol^−1^ K^−1^, and ΔG = 4.34 kcal/mol at 298 K. As expected, ΔG° approached zero near T_m_, consistent with the equilibrium between native and denatured states. These results confirm that butein modestly stabilizes DNA compared to EB, which shows stronger intercalative stabilization.

### 2.5. Effect of Urea on Butein–DNA Binding

The urea’s ability to destabilize DNA double helix is also used to probe the binding mode of drugs with DNA. When intercalating molecules are present, adding urea forces the intercalators out of DNA helix. This release alters the drug’s fluorescent properties. In contrast, groove binders show minimal changes in fluorescence behaviour of drugs upon urea treatment [22]. In this study, we employed the urea disruption assay to investigate butein’s binding to DNA (Figure 4B). We observed that adding urea to the DNA–butein complex did not produce much change in the fluorescence. This finding suggests that butein likely interacts at the groove of DNA rather than intercalation. Similar finding is documented earlier where addition of urea to the DNA–capsaicin complex did not alter fluorescence of the DNA–capsaicin system, indicating that binding of capsaicin to DNA is non-intercalative [25].

### 2.6. Effect of Butein on DNA Viscosity 

Further examination of DNA and butein interaction was conducted using viscosity measurements. This technique is highly sensitive to changes in DNA length, making it a valuable tool to distinguish between intercalative and non-intercalative binding of small molecules to DNA [18]. Molecules that intercalate between DNA base pairs cause a noticeable increase in DNA’s viscosity. This rise is attributed to base pair separation at intercalation site, effectively lengthening the DNA molecule [26]. Conversely, groove binders or the molecules interacting electrostatically with DNA have minimal impact on DNA’s viscosity [27]. The plot (Figure 4C) depicts the relative viscosity (η/η_0_) vs. ratio of butein to DNA concentration. Notably, this plot reveals negligible changes in DNA viscosity with the increase in butein concentration. This observation further supports a non-intercalative binding mode, aligning with thermal denaturation and urea disruption experiments. A previous study similarly reported only a small change in DNA viscosity following glipizide addition, consistent with non-intercalative binding [28].

### 2.7. Identification of DNA-Binding Site 

Competitive displacement assays use fluorescent dyes with established DNA-binding modes to investigate ligand-binding sites. A decrease in the fluorescence of a preformed DNA–dye complex following ligand addition may indicate competition for overlapping binding sites [29].

To study the binding site, we employed a competitive displacement assay using ethidium bromide (EB) [30]. EB is a well-established intercalating agent that exhibits weak fluorescence alone. However, upon intercalating between DNA base pairs, its fluorescence intensity significantly increases [30]. If a drug binds via intercalation, it should displace EB from DNA, leading to a decrease in fluorescence intensity of the EB-DNA complex. The extent of decrease in fluorescence reflects the drug’s intercalation strength [31,32]. The addition of butein to the DNA-EB complex did not cause any significant change in fluorescence intensity (Supplementary Figure S1A). This observation aligns with previous experiments that butein likely interacts with DNA through a non-intercalative mode.

To further exclude the possibility of butein being DNA intercalator, AO competitive displacement assay was employed [33]. Similar to EB, AO is a well-known intercalating agent with weak intrinsic fluorescence. However, upon intercalating between DNA base pairs, its fluorescence intensity rises many folds [33]. If butein intercalates into DNA, it should compete with AO for same binding site, decreasing the fluorescence of the DNA-AO complex. The observed slight change in fluorescence with the increase in butein concentrations to the DNA-AO complex provides further validation of non-intercalative mode of butein interaction (Supplementary Figure S1B).

Hoechst 33258 is a minor-groove binder whose fluorescence increases upon DNA binding [34,35]. Increasing butein concentration substantially decreased the fluorescence intensity of the DNA–Hoechst complex (Supplementary Figure S1C), consistent with competition for minor-groove binding sites.

**Figure 5:**
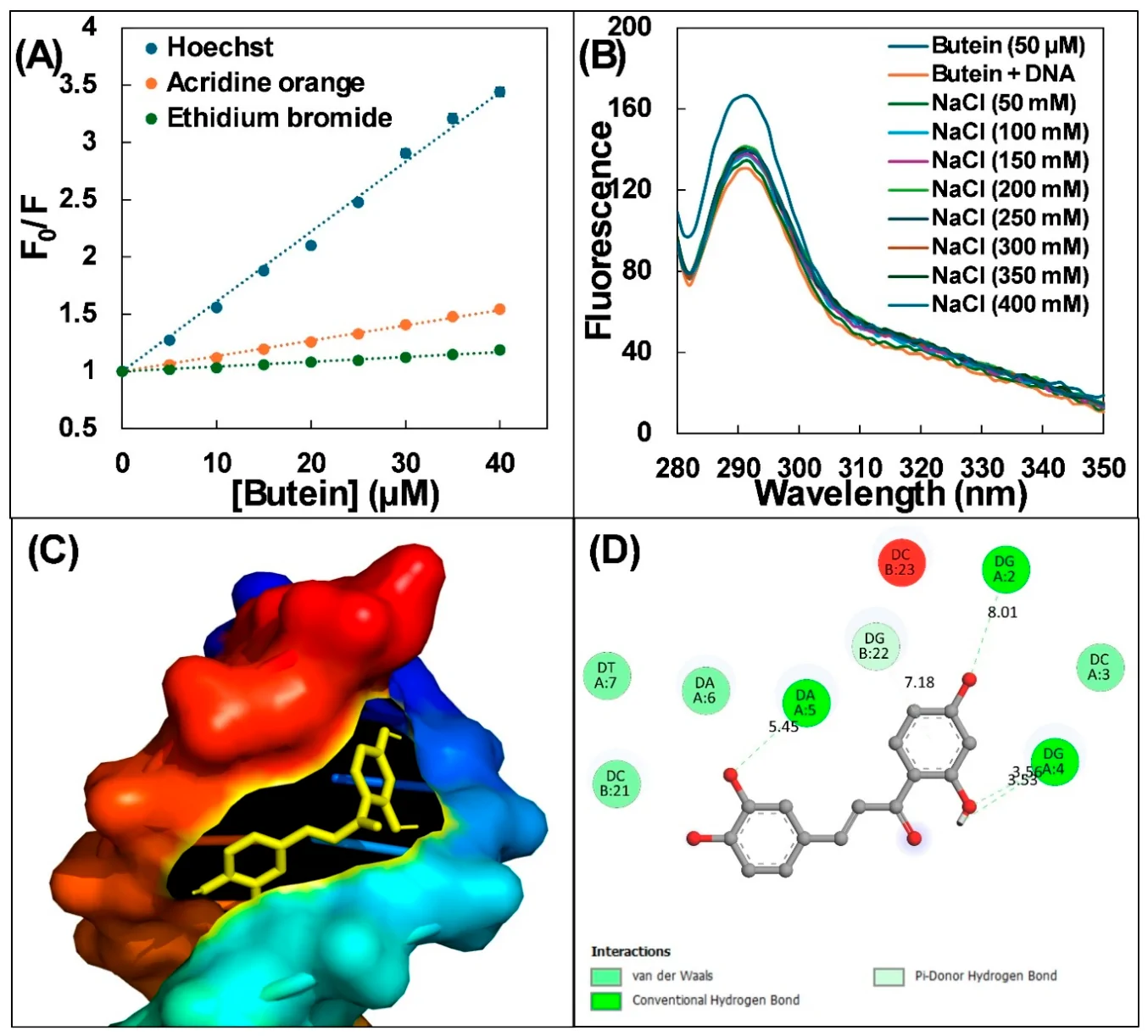
(**A**) Stern–Volmer plot for dye displacement assays. DNA = 15 µM; dye = 5 µM; butein = 5, 10, 15, 20, 25, 30, 35, and 40 µM. (**B**) Effect of NaCl on DNA–butein complex. Butein = 50 µM; DNA–butein complex = 50 µM each, NaCl = 50, 100, 150, 200, 250, 300, 350, and 400 mM. (**C**) Docked pose of DNA–butein complex. Butein interacts at the minor groove of DNA. (**D**) Two-dimensional representation of DNA–butein complex.

To quantitatively compare the displacement effects of dyes, we employed the Stern–Volmer equation to calculate K_sv_ values. The Stern–Volmer plots are presented in Figure 5A. The K_sv_ value measured for Hoechst (6.10 ± 0.04 × 10^4^ M^−1^) was significantly higher than those observed for acridine orange (1.34 ± 0.01 × 10^4^ M^−1^) and ethidium bromide (0.42 ± 0.09 × 10^4^ M^−1^). Furthermore, the percent dye displacement was also calculated as shown in Supplementary Figure S1D. Butein produced the greatest apparent displacement of Hoechst 33258 (70.93%), followed by AO (35.20%) and EB (15.81%). Together with the thermal denaturation, urea disruption, and viscosity results, these findings support preferential binding in the DNA minor groove.

### 2.8. Effect of Ionic Strength on Butein– DNA Binding

The involvement of electrostatic interactions in DNA–butein complex formation was investigated with the increase in ionic strength using NaCl. Groove binding and intercalative binding modes typically involve the small molecule being positioned near the DNA double helix, but not inside the DNA helix. Conversely, electrostatic interactions might occur from a distance, outside the DNA helix itself [36]. If electrostatic interactions are involved in DNA–butein complex formation, increasing concentration of NaCl (which disrupts electrostatic interactions) should lead to release of butein from DNA, causing rise in fluorescence intensity. As depicted in Figure 5B, negligible changes in fluorescence were observed with the increase in NaCl concentration from 50 to 400 mM, suggesting that salt-sensitive electrostatic interactions were not a major determinant of binding under the experimental conditions. A similar response has been reported for the capsaicin–DNA complex [25].

### 2.9. Molecular Docking of Butein with DNA

Molecular docking offers valuable insights into ligand–receptor interactions [37]. This tool can generate detailed information about DNA–drug interactions. Molecular docking not only allows for modelling of ligand flexibility but also utilizes complex molecular mechanics for calculation of binding energy [37]. Figure 5C shows docked conformation of butein bound to DNA [38]. The analysis of only the lowest energy docked pose was performed. It was found that butein was situated within minor groove of DNA dodecamer. Notably, docked complex was stabilized by various non-covalent interactions. Four hydrogen bonds involving dG2, dG4, and dA5 were identified, together with van der Waals contacts involving dT7, dC21, dA6, and dC3 (Figure 5D). The docking score was −7.4 kcal mol^−1^. Because docking scores and experimentally derived binding free energies are obtained using different approaches, they should not be interpreted as directly equivalent. AutoDock Vina-1.1.2 treated DNA as rigid during docking; subsequent molecular dynamics simulations were used to examine the complex with DNA flexibility included.

### 2.10. Molecular Dynamics Simulations of DNA–butein complex

To examine the dynamics and stability of DNA–butein complex, we simulated the complex at physiological conditions. The lowest energy docked complex was used as the initial structure for MD simulations. DNA alone was also simulated that served as the control. The findings from MD simulations are discussed below.

#### 2.10.1. Examination of Deviation and Fluctuations

**Figure 6:**
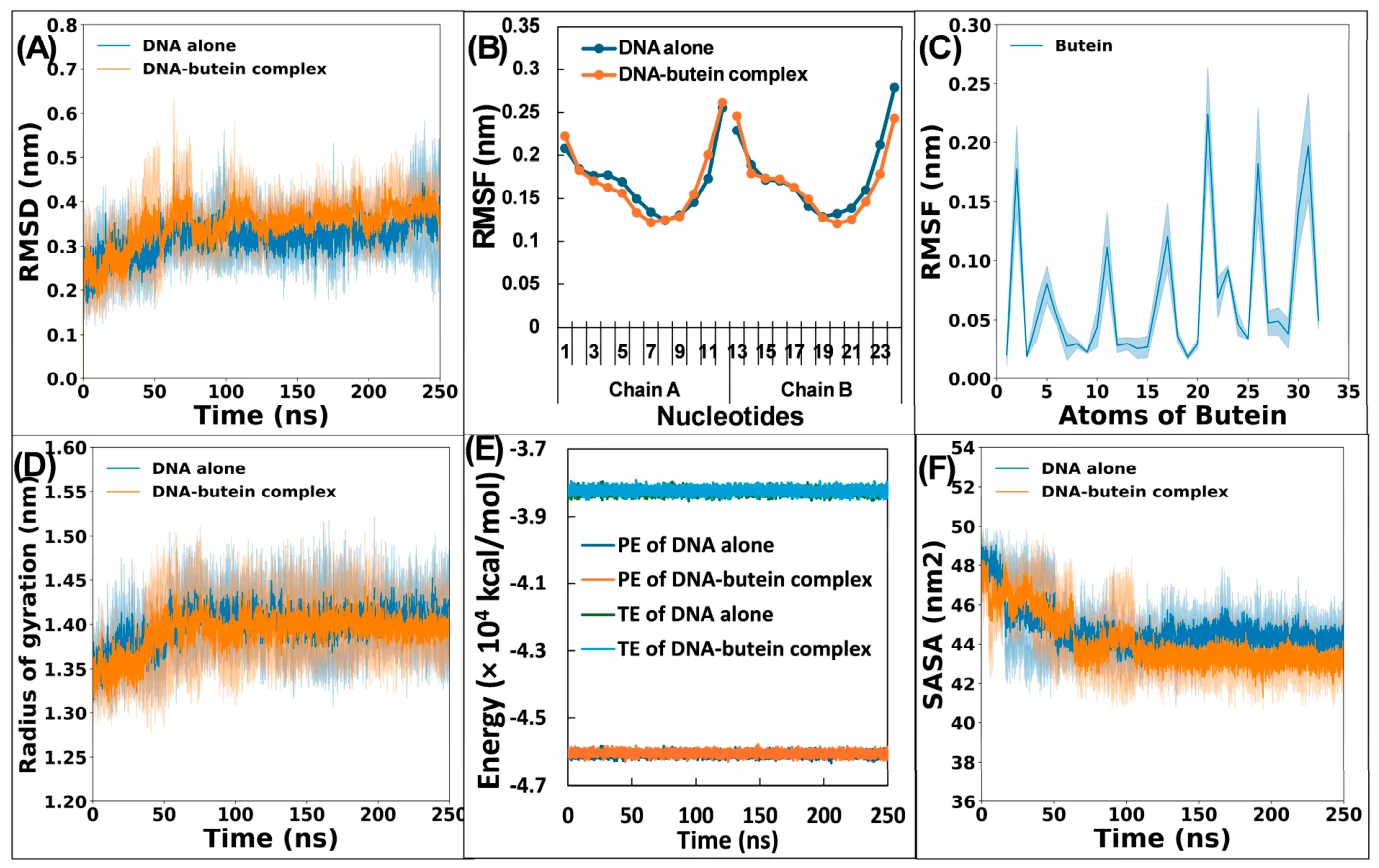
(**A**) Root-mean-square deviations of DNA alone and DNA–butein complex over 250 ns simulation. (**B**) Average root-mean-square fluctuations (RMSF) of nucleotides of DNA in absence and presence of butein. (**C**) Average RMSF of atoms of butein. (**D**) Radius of gyration (R_g_) of DNA alone and DNA–butein complex over 250 ns simulation. (**E**) Potential energy (PE) and total energy (TE) of DNA alone and DNA–butein complex over 250 ns simulation. (**F**) Solvent accessible surface area (SASA) of DNA alone and DNA–butein complex over 250 ns simulation.

To assess the stability of DNA alone and in complex with butein, we calculated the root-mean-square deviations (RMSD) for each system. RMSD measures structural deviation from a reference structure over time. The RMSD was calculated with respect to the initial structures. Lower RMSD values indicate smaller deviations from the reference structure. Figure 6A depicts the RMSD profiles for both DNA and DNA–butein complex. The RMSD profiles of both systems showed that free DNA and DNA–butein complex show some initial deviations roughly until 80 ns before reaching a plateau. This suggests that the systems require this initial period to reach a state of equilibrium. The average RMSD for free DNA was found to be 0.32 ± 0.04 nm, which is consistent with a previous report [28]. The DNA–butein complex also displayed an average RMSD of 0.35 ± 0.05 nm. Interestingly, binding of butein did not have much effect on RMSD of DNA. This observation also aligns with previous studies, where glipizide binding had little effect on DNA RMSD values [28].

To gain further insights into the dynamic behaviour of nucleotides of DNA, we calculated the root-mean square fluctuations (RMSF) for each nucleotide. RMSF values reflect the average fluctuation of nucleotides relative to their initial positions during the simulation. Lower RMSF values indicate greater stability of overall structure. As observed in Figure 6B, most nucleotides exhibited fluctuations below 0.20 nm, implying overall structural stability of DNA in both free and butein-bound states. However, some spikes in the RMSF were observed for terminal nucleotides in both DNA chains, the reason being inherent flexibility of these terminal regions in aqueous environments [24]. To assess the dynamic nature of butein with DNA, RMSF of its atoms was computed (Figure 6C). The observed fluctuations in butein’s RMSF profile indicate a degree of mobility at the binding site. This mobility could be attributed to presence of rotatable bonds within the butein molecule and its conformational adjustments within the minor groove.

#### 2.10.2. Examination of DNA’s Compactness and Physicochemical Parameters

The radius of gyration (R_g_) was used to assess DNA compactness during the MD simulations. Figure 6D depicts the R_g_ values for DNA alone and the DNA–butein complex. Apart from a minor initial decrease, both systems displayed stable R_g_ values throughout simulation trajectory. The data suggest that butein binding does not significantly alter the overall compactness of DNA, further supporting the stability of complex. To gain deeper insights into the complex’s stability, we also analysed potential and total energies (Figure 6E). The DNA–butein complex maintained uniform potential energy, like those of free DNA. Similarly, total energy profiles for both systems remained consistent throughout the simulation. The analysis of these parameters indicates that the DNA–butein complex likely remains stable under physiological conditions.

Solvent accessible surface area (SASA) is another metric employed to assess stability of a complex in solution. SASA reflects the surface area of a molecule accessible to the solvent. Figure 6F shows SASA of DNA alone and DNA–butein complex. The average SASA for free DNA was 44.72 ± 1.01 nm^2^, while the DNA–butein complex showed an average SASA of 44.11 ± 1.29 nm^2^. Notably, the complex exhibited minimal changes in SASA compared to free DNA. Moreover, a slight decrease in the SASA shows that solvent accessibility to DNA was decreased after complexation of butein. For comparison, a previous study reported average SASA values nearly 45 nm^2^ for both free DNA and DNA–glipizide complex during simulation [28].

#### 2.10.3. Hydrogen Bond Analysis

**Figure 7:**
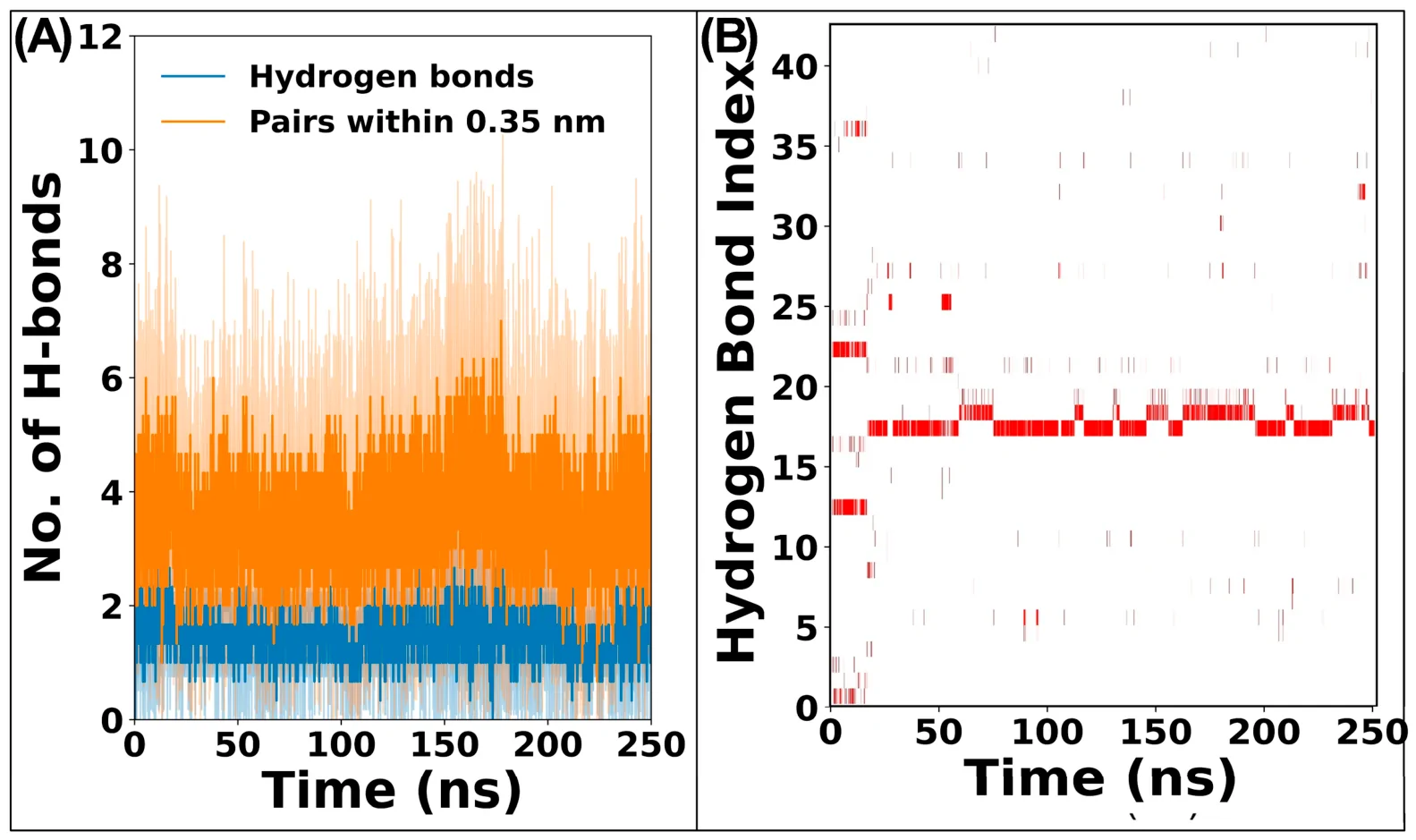
(**A**) Number of hydrogen bonds formed between DNA and butein over 250 ns simulation. (**B**) Hydrogen bond existence map of DNA–butein complex over 250 ns simulation.

To gain deeper understanding, we investigated hydrogen bond formation. Figure 7A depicts number of hydrogen bonds formed between butein and DNA during simulation. The DNA–butein complex displayed an average of 1.50 ± 0.36 hydrogen bonds per frame. This observation aligns well with molecular docking, which predicted the presence of hydrogen bonds between butein and DNA. The hydrogen bond existence map shows the presence of dynamic hydrogen bonds between DNA and butein (Figure 7B). The fluctuating nature of hydrogen bond profile suggests dynamic hydrogen bonds between butein and DNA. The interactions of water molecules near the binding site also affect the profile of the hydrogen bond [39]. Moreover, the presence of rotatable bonds within butein also allows for conformational adjustments, potentially leading to formation and breakage of hydrogen bonds over time. Water molecules present in simulation system can interact with both butein and binding site nucleotides, influencing the hydrogen bonds between drug and DNA [28,39]. To quantify the persistence of hydrogen bond formation, we calculated hydrogen bond occupancy for each hydrogen bond pair. In the DNA–butein complex, thymine residue at position 7 (dT7) exhibited the highest occupancy (51.3%), indicating a frequent hydrogen bond with butein. Similarly, dT8 also showed good occupancy of hydrogen bonds. Other nucleotides were also found to be involved in hydrogen bonding; however, their occupancies were lower. The finding aligns with a previous report where dG10 showed the highest occupancy (45.8%) in the glipizide and DNA interaction [28].

#### 2.10.4. Principal Component Analysis

**Figure 8:**
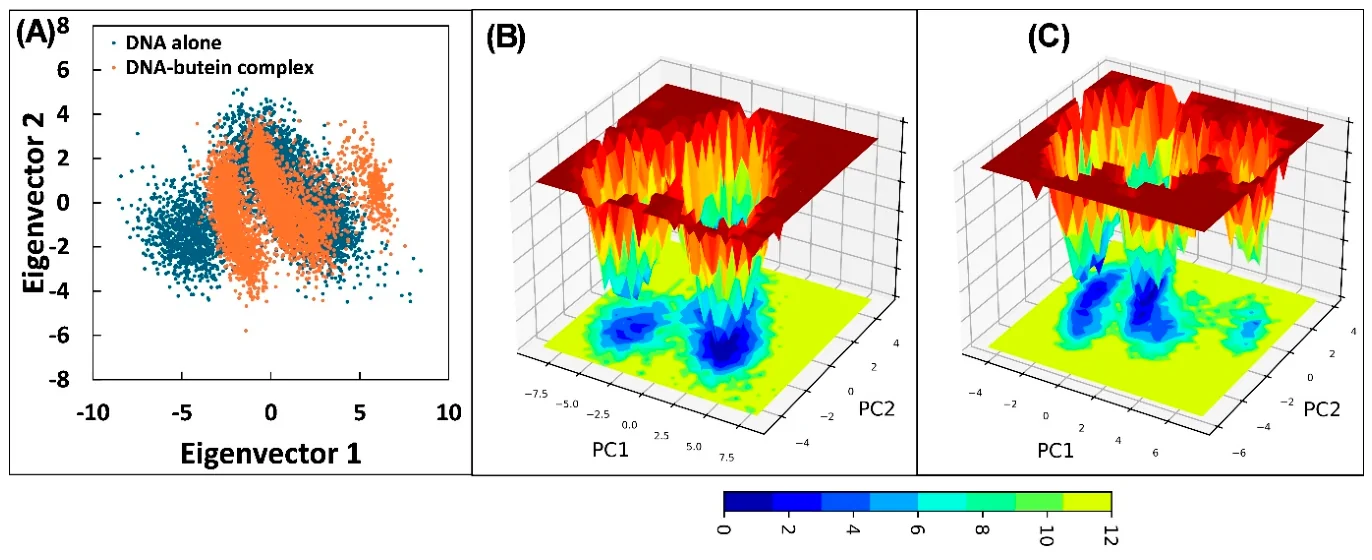
(**A**) Projection of eigenvectors of DNA alone and DNA–butein complex in two dimensions. (**B**) Free energy landscape of DNA alone. (**C**) Free energy landscape of DNA–butein complex.

Principal component analysis (PCA) was used to examine the collective motions of DNA in its free and butein-bound states. PCA reduces the complexity of large datasets into a smaller set of informative components called eigenvectors. These eigenvectors represent the most significant collective motions within the molecule. We utilized PCA to explore the flexibility of DNA in both its free and butein bound states. The analysis involved construction of covariance matrix based on the atomic fluctuations during simulations. This matrix was then diagonalized to obtain a set of eigenvalues and eigenvectors. Eigenvalues reflect the magnitude of corresponding eigenvector, with higher values indicating more significant collective motions. The projection of eigenvectors in two dimensions is shown in Figure 8A. The 2D projection revealed that the DNA–butein complex occupied a slightly lesser conformational space compared to free DNA. The finding suggests that butein binding might lead to a minor decrease in DNA flexibility. Free energy landscapes were also constructed using eigenvector data. These landscapes represent the energetically favourable conformations that a molecule can adopt. The free energy landscape is shown in Figure 8B,C. Trajectories of both DNA alone and DNA–butein complex reached energy minima within their respective landscapes. However, the positions of these minima differed between the two systems. In both systems, three energy minima points were obtained. In DNA alone simulation, energy minima were obtained at 76.25, 112.45, and 230.55 ns. Similarly, in DNA–butein complex, the energy minima were obtained at 162.00, 217.40, and 217.60 ns, respectively. Overall, PCA analysis suggests that the butein interaction does not significantly alter the overall structure or flexibility of DNA, while a slight decrease in flexibility was observed upon butein complexation.

#### 2.10.5. Examination of Binding Energies

**Figure 9:**
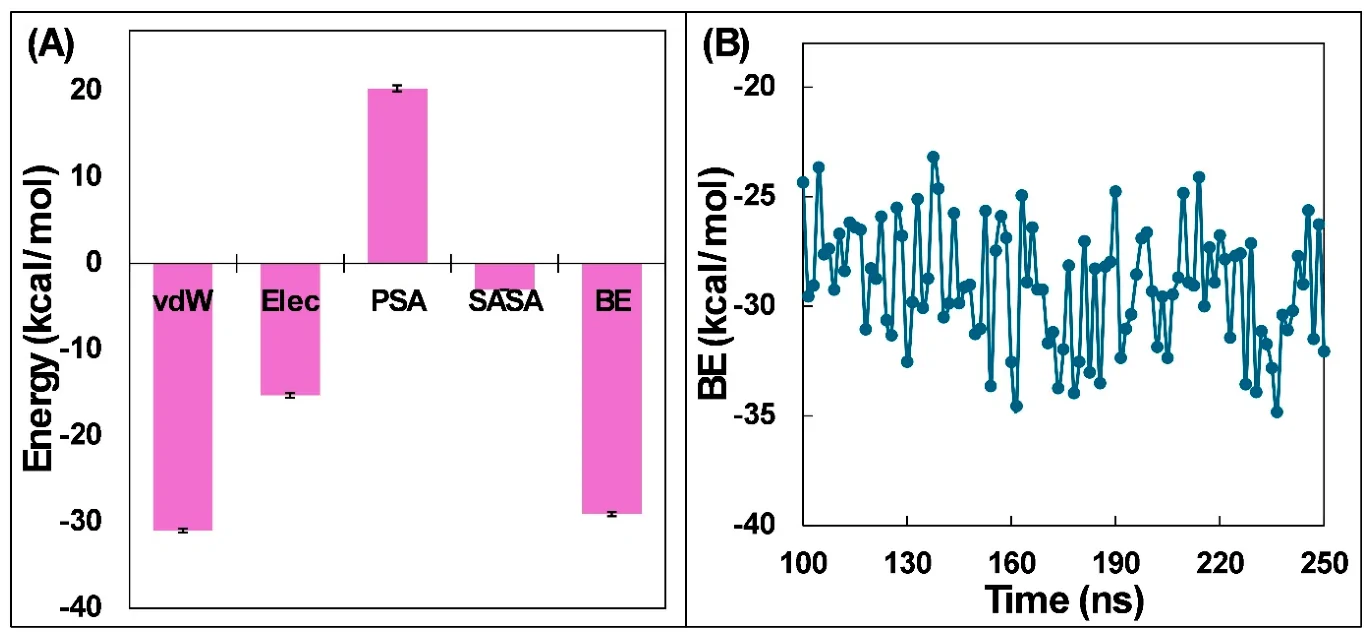
(**A**) MM-PBSA binding energy contribution of various energies for DNA–butein complex. vdW is van der Waals energy; Elec is electrostatic energy; PSA is polar solvation energy; SASA is SASA energy; and BE is overall binding energy. (**B**) Energy per frame for DNA–butein complex from 100 to 250 ns.

MM-PBSA calculations were used to estimate the energetic contributions to butein–DNA binding. One hundred frames were extracted at regular intervals from the 100–250 ns portion of the complex trajectory. These frames were then used to estimate the different energy components. It is well-established that interactions between DNA and drugs are primarily driven by non-covalent forces, including hydrophobic forces, van der Waals forces, hydrogen bonding, and electrostatic interactions [40]. Each of these forces can contribute either favourably (negative energy values) or unfavourably (positive energy values) to the overall binding affinity. The MM-PBSA analysis revealed that van der Waals forces exerted the strongest favourable influence (Figure 9A). Electrostatic forces were also found to play a significant role in stabilizing the complex. The contribution of SASA energy was minimal. Interestingly, polar solvation energy showed positive value, indicating that it counteracts the favourable interactions. The calculated overall energy for DNA–butein complex was −33.15 ± 3.58 kcal/mol. This value signifies a favourable interaction between the two molecules.

The MM-PBSA data also yielded energetic contributions of individual nucleotides of DNA. Table 4 summarizes the nucleotides exhibiting the most significant energy contributions. The analysis revealed that dT8, dT20, dC9, dT19, dC21, dT7, dA5, dA6, dG22, and dA18 played a main role in the overall binding affinity. Interestingly, the polar energies for many of these high-energy residues were positive. This suggests that their polar interactions hinder the binding process. However, the overall negative values of total energy for these residues indicate that they still contribute favourably to DNA–butein complex formation. The MM-PBSA data were also used to calculate binding energy on a per-frame basis (Figure 9B). As expected, the overall binding energy exhibited some fluctuations during simulation. It is important to note that these fluctuations are well within acceptable range for this type of analysis. The minimum and maximum overall binding energies obtained from MM-PBSA calculations were −35.72 kcal/mol and −26.17 kcal/mol, respectively. Such variations in overall binding energy also indicate the dynamic nature of the DNA–butein complex during the simulation.

## 3. Materials and Methods

### 3.1. Materials and Reagents

In this study, calf thymus DNA was obtained from Sigma Aldrich (St. Louis, MO, USA). Calf thymus DNA was selected as it is one of the most widely used models in drug–DNA binding assays and its physicochemical and spectral properties are well documented. Hoechst 33258 and AO (acridine orange) were also purchased from Sigma Aldrich. Butein (B3803) was obtained from Tokyo Chemical Industry Pvt. Ltd. (Chennai, India).

### 3.2. Preparation of Stock Samples

A calf thymus DNA stock solution (2 mg mL^−1^) was prepared by dissolving 60 mg of DNA in 30 mL of Tris–HCl buffer (10 mM, pH 7.4) and incubating it overnight at 4 °C. The solution was heated at 95 °C for 5 min and slowly cooled to room temperature to promote reannealing. Gentle mixing was used to obtain a homogeneous solution. DNA concentration was determined from absorbance at 260 nm using a molar absorptivity of 6600 M^−1^ cm^−1^ [41]. The A_260_/A_280_ ratio was 1.86, indicating low protein contamination. The stock solution was stored at −20 °C. Butein was dissolved in DMSO to prepare a 10 mM stock solution and subsequently diluted with Tris–HCl buffer. The final DMSO concentration was below 0.5% (*v*/*v*).

### 3.3. UV-Visible Spectroscopy

Shimadzu 1800 UV-VIS Spectrophotometer (Shimadzu, Kyoto, Japan) was employed to study the interaction between butein and DNA. The UV–visible spectra of both butein alone and DNA–butein complex were acquired in range of 230–530 nm. In this experiment, a fixed concentration of butein (20 μM) was taken and concentration of DNA was increased from 5 to 35 μM. Baseline correction was performed using Tris-HCl buffer (10 mM, pH 7.4). In another experiment, absorption spectra of DNA alone at various concentrations (5 to 35 μM) were taken. The final UV–visible spectra were obtained by subtracting the UV–visible spectra of DNA alone from that of the DNA–butein complex at the respective concentrations.

### 3.4. Steady-State Fluorescence

Fluorescence spectroscopy was also employed to further investigate the interaction between butein and DNA. The fluorescence was measured using Shimadzu RF-5301PC spectrophotometer. A four-way cuvette with a 10 mm path length was used for all fluorescence measurements. The fluorescence emission spectra of butein were recorded in the emission range of 280–350 nm by exciting at 260 nm. Since DNA also absorbs at 260 nm, the observed fluorescence was corrected for inner-filter effects. Butein concentration was fixed at 30 μM, and DNA concentration was varied from 10 to 80 μM. Measurements were performed at 293, 298, 303, and 310 K to evaluate the thermodynamic parameters associated with DNA–butein complex formation. To confirm the photostability of butein, UV-vis spectra of butein were recorded before and after excitation at 260 nm. No noticeable spectral changes were observed, indicating that butein remained photostable.

### 3.5. DNA Melting Assays

For DNA melting assays, DNA’s (50 μM) absorbance was taken at different temperatures in the range of 25–95 °C at 260 nm. The same experiment was performed for the DNA–butein complex (50 μM each) where changes in T_m_ (melting temperature) was determined. For the positive control, the EB-DNA complex (complex of DNA with ethidium bromide) was used. In each case, the ratio of absorbances recorded at a temperature of 25 °C was plotted against the temperature. The midpoint of this curve taken as the T_m_ value.

### 3.6. Effect of Urea on DNA–Butein Complex

A mixture containing DNA and butein at 50 μM each was titrated with urea over a final concentration range of 0–1.2 M. Samples were excited at 260 nm, and fluorescence emission spectra were recorded over 280–350 nm.

### 3.7. Viscosity Measurements

Viscosity measurements were performed using an Ubbelohde viscometer in a water bath maintained at 25 °C. DNA concentration was fixed at 100 μM, and butein concentration ranged from 20 to 100 μM. Flow time was measured three times at each concentration, and the mean value was used for analysis. Relative viscosity, expressed as (η/η_0_)^1/3^, was plotted against the butein-to-DNA concentration ratio. In this case, η_0_ and η represent the viscosity of DNA solutions without and with butein, respectively.

### 3.8. Competitive Displacement Assays

The competitive binding assays were conducted using different fluorescent dyes. These dyes can be broadly categorized into two groups: intercalating dyes that insert themselves between DNA base pairs, and groove-binding dyes that interact at the grooves of the DNA helix. In the ethidium bromide (EB) displacement assay, a fixed DNA-EB complex (15 μM DNA and 5 μM EB) was titrated with increases in butein concentrations. The emission signals of this complex were measured between 500 and 700 nm, with excitation at 476 nm. Similar competitive binding assays were performed using other site-specific dyes, i.e., acridine orange (AO) and Hoechst 33258. For AO displacement, the emission of the DNA-AO complex was recorded from 500 to 700 nm with excitation at 480 nm. In Hoechst 33258 assay, excitation was conducted at 343 nm, and emission was recorded within the range from 350 to 600 nm. All fluorescence measurements were conducted at 25 °C and the excitation and emission slit widths were set to 5 nm. The fluorescence emission was recorded at 1 nm intervals with scan speed of 120 nm/min.

### 3.9. Effect of Ions on DNA–Butein Complex

This experiment was conducted to investigate the influence of ions on DNA–butein complex formation. A fixed concentration of DNA–butein complex (50 μM each) was titrated with varying concentrations of NaCl (50–400 mM). The emission spectra were recorded between 280 and 350 nm with excitation at 260 nm. This assay was conducted in Tris-HCl buffer (10 mM, pH 7.4).

### 3.10. Molecular Docking

To investigate the interaction at the molecular level, we employed AutoDock Vina for molecular docking [38]. This software is known for its accuracy and speed [42]. The 3D structure of B-form DNA d(CGCGAATTCGCG)_2_ dodecamer was retrieved from the PDB (ID: 1BNA). Crystallographic water molecules were removed before docking. Non-polar hydrogen atoms were added, and Kollman charges were assigned using the AutoDockTools-1.5.6 [43]. A grid box measuring 26 × 28 × 44 Å was defined to encompass the DNA molecule. This grid serves as the search space for potential butein binding sites. The DNA structure was saved in the PDBQT format, the file format required for AutoDock Vina. The butein structure (CID: 5281222) was taken from the PubChem database in SDF format. This file was then converted to the PDB format. The binding mode of butein with the DNA molecule exhibiting the lowest energy was analysed using Discovery Studio 2024 and PyMOL-3.1.6.1.

### 3.11. Molecular Dynamics Simulations

To further explore the interaction of butein with DNA, MD simulations were performed with the Gromacs 2018.1 software [44]. The simulations were performed to examine the stability and dynamics of the butein–DNA complex. First, topology files were generated. The *pdb2gmx* tool of Gromacs was used for DNA’s topology preparation, while AmberTools21’s Antechamber package was used to prepare the topology for butein [45]. Amber99sb-ILDN force field was used to simulate both systems [46]. DNA and butein topologies were then merged to create the topology of complex. A control simulation of DNA alone was also performed to compare with the DNA–butein complex. To mimic the physiological environment, water molecules of the TIP3P model were added, and then 22 chlorine ions were placed to neutralize the overall system. Also, 150 mM NaCl was added to match physiological salt levels. The system’s energy was minimized, followed by a two-step equilibration process. The first equilibration phase (NVT ensemble) employed the V-rescale thermostat for 1 ns at 310 K [47], while subsequent equilibration (NPT ensemble) utilized the Parrinello–Rahman barostat for another 1 ns at 1 bar [48]. A 250 ns production simulation was subsequently performed for each system. After the simulations, PBC corrections were applied. With the g_mmpbsa-1.1.0 package, binding energy between DNA and butein was calculated [49].

### 3.12. Statistical Analysis

All experiments were performed in three independent replicates. Results are presented as mean ± standard deviation, and error bars indicate standard deviations.

## 4. Conclusions

This study characterized the interaction between butein and calf thymus DNA using experimental and computational approaches. Fluorescence-derived binding constants were in the order of 10^3^ M^−1^, indicating weak to moderate affinity. Thermodynamic analysis indicated spontaneous binding that was both enthalpically and entropically favourable. DNA melting, urea disruption, viscosity, and competitive displacement assays supported preferential binding in the minor groove. Molecular docking identified hydrogen bonds and van der Waals contacts, while molecular dynamics simulations suggested a stable complex with limited changes in DNA structure over 250 ns at 310 K. These findings provide a molecular basis for further investigation of butein–DNA interactions. Further studies are needed to establish whether this binding contributes to butein’s biological effects.

## Figures and Tables

**Table 1:** The Stern–Volmer constants (K_sv_), quenching rate constants (K_q_), and correlation coefficients (R^2^) obtained from steady-state fluorescence measurements of butein and DNA interaction at different temperatures.

Temp (°C)	K_sv_ (10^3^ M^−1^)	R^2^	K_q_ (10^11^ M^−1^ s^−1^)
20	4.31 ± 0.14	0.9976	4.31 ± 0.14
25	3.77 ± 0.22	0.9963	3.77 ± 0.22
30	3.11 ± 0.22	0.9969	3.11 ± 0.22
37	2.56 ± 0.20	0.9943	2.56 ± 0.20

**Table 2:** Number of binding sites (n) and binding constant (K) obtained using steady-state fluorescence for the interaction of butein with DNA at different temperatures.

Temp (°C)	K (×10^3^ M^−1^)	n	R^2^
20	5.62 ± 0.34	1.03 ± 0.01	0.9990
25	5.19 ± 0.24	1.03 ± 0.01	0.9955
30	4.68 ± 0.24	1.04 ± 0.01	0.9978
37	4.04 ± 0.31	1.05 ± 0.02	0.9944

**Table 3:** Various thermodynamic parameters obtained using steady-state fluorescence for interaction of butein with DNA at different temperatures.

Temp (°C)	ΔG (kcal mol^−1^)	ΔH (kcal mol^−1^)	ΔS (cal mol^−1^ K^−1^)	R^2^
20	−5.03 ± 0.22	−3.54 ± 0.20	5.09 ± 0.19	0.9948
25	−5.06 ± 0.19	−3.54 ± 0.20	5.09 ± 0.19	
30	−5.08 ± 0.24	−3.54 ± 0. 20	5.09 ± 0.19	
37	−5.12 ± 0.16	−3.54 ± 0. 20	5.09 ± 0.19	

**Table 4:** Total energy, apolar energy, and polar energy (kcal/mol) contribution of major energy contributing nucleotides of DNA for complexation of butein.

S. No.	Residues	Polar Energy	Apolar Energy	Total Energy
1	dT8	0.96 ± 0.08	−0.19 ± 0.01	−2.76 ± 0.10
2	dT20	0.55 ± 0.10	−0.19 ± 0.01	−2.07 ± 0.08
3	dC9	−0.46 ± 0.06	−0.14 ± 0.01	−2.05 ± 0.08
4	dT19	1.11 ± 0.07	−0.15 ± 0.01	−1.88 ± 0.07
5	dC21	−0.10 ± 0.08	−0.11 ± 0.01	−1.87 ± 0.07
6	dT7	1.47 ± 0.09	−0.14 ± 0.01	−1.63 ± 0.12
7	dA5	0.47 ± 0.04	−0.07 ± 0.01	−1.41 ± 0.07
8	dA6	0.44 ± 0.07	−0.08 ± 0.01	−1.41 ± 0.06
9	dG22	−0.07 ± 0.06	−0.07 ± 0.01	−1.32 ± 0.06
10	dA18	0.61 ± 0.05	−0.06 ± 0.01	−1.02 ± 0.06

## Data Availability

The original contributions presented in this study are included in the article/Supplementary Materials. Further inquiries can be directed to the corresponding author.

## Supplementary Materials

The following supporting information can be downloaded at: https://www.mdpi.com/article/10.3390/molecules31203750/s1. Figure S1: (A) Fluorescence titration of DNA-EB complex with increases in the concentration of butein. (B) Fluorescence titration of DNA-AO complex with increasing concentration of butein. (C) Fluorescence titration of the DNA–Hoechst complex with increases in the concentration of butein. (D) Percent displacement of EB, AO, or Hoechst by butein.
