# Supplementary material for: Butein Interacts at the Minor Groove of DNA: Multi-Spectroscopic, Thermodynamic, and Bioinformatic Analyses
Source: Molecules. 2026 Oct 19;31(20):3750. doi: 10.3390/molecules31203750 (PMC13707773; doi:10.3390/molecules31203750)
Supplement: Supplementary file 1 [file molecules-31-03750-s001.zip › molecules-4582746-supplementary.pdf]

Butein interacts at the minor groove of DNA: Multi-spectroscopic, thermodynamic, and bioinformatic analysis

Supplementary Figure

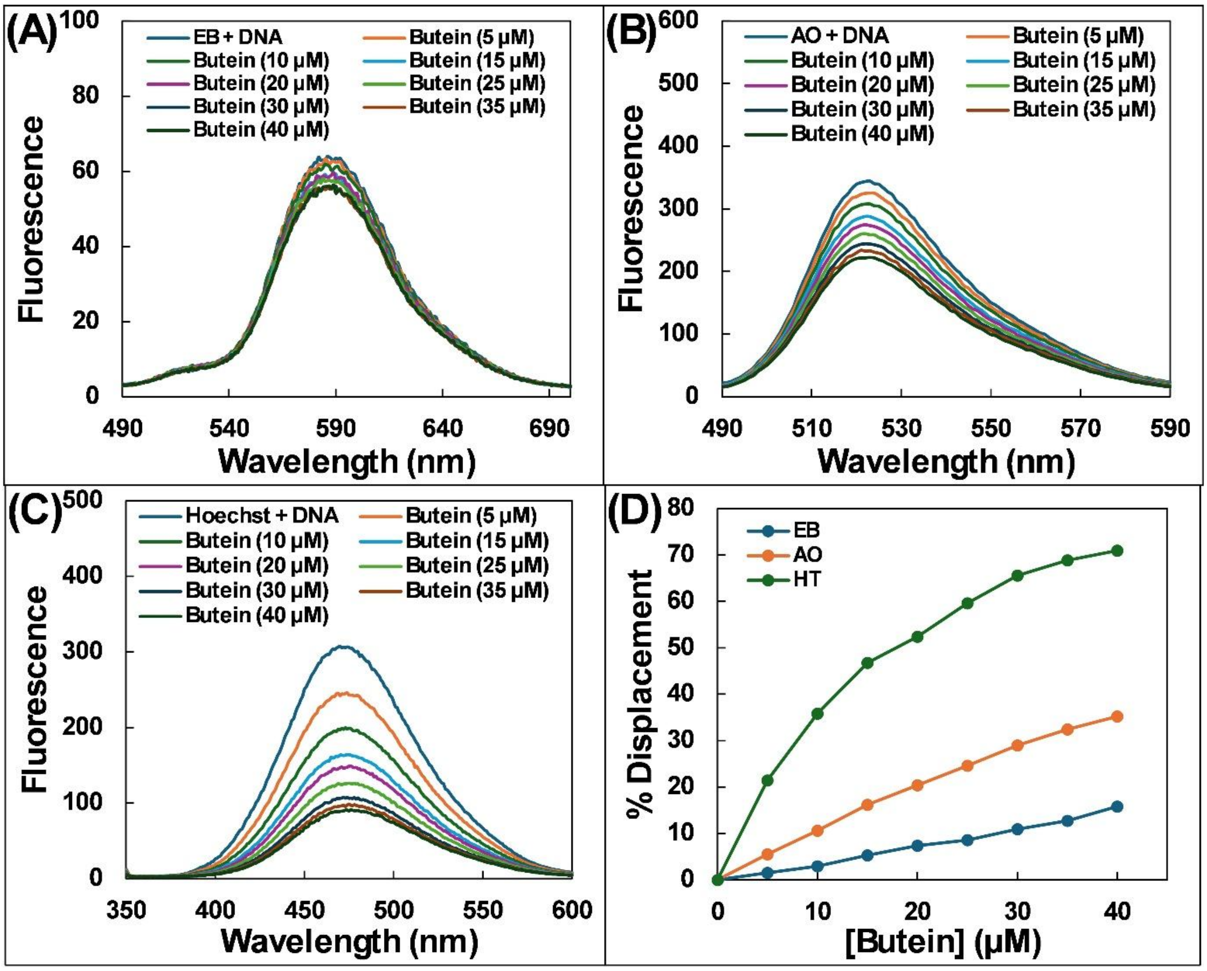

`**Supplementary Fig S1**. (A) Fluorescence titration of DNA-EB complex with increasing concentration of butein. (B) Fluorescence titration of DNA-AO complex with increasing concentration of butein. (C) Fluorescence titration of DNA-Hoechst complex with increasing concentration of butein. (D) Percent displacement of EB, AO, or Hoechst by butein.
